# Supplementary figures and images for: Transcriptome analyses of Anguillicola crassus from native and novel hosts
Source: PeerJ. 2014 Nov 27;2:e684. doi: 10.7717/peerj.684 (PMC4250067; doi:10.7717/peerj.684)

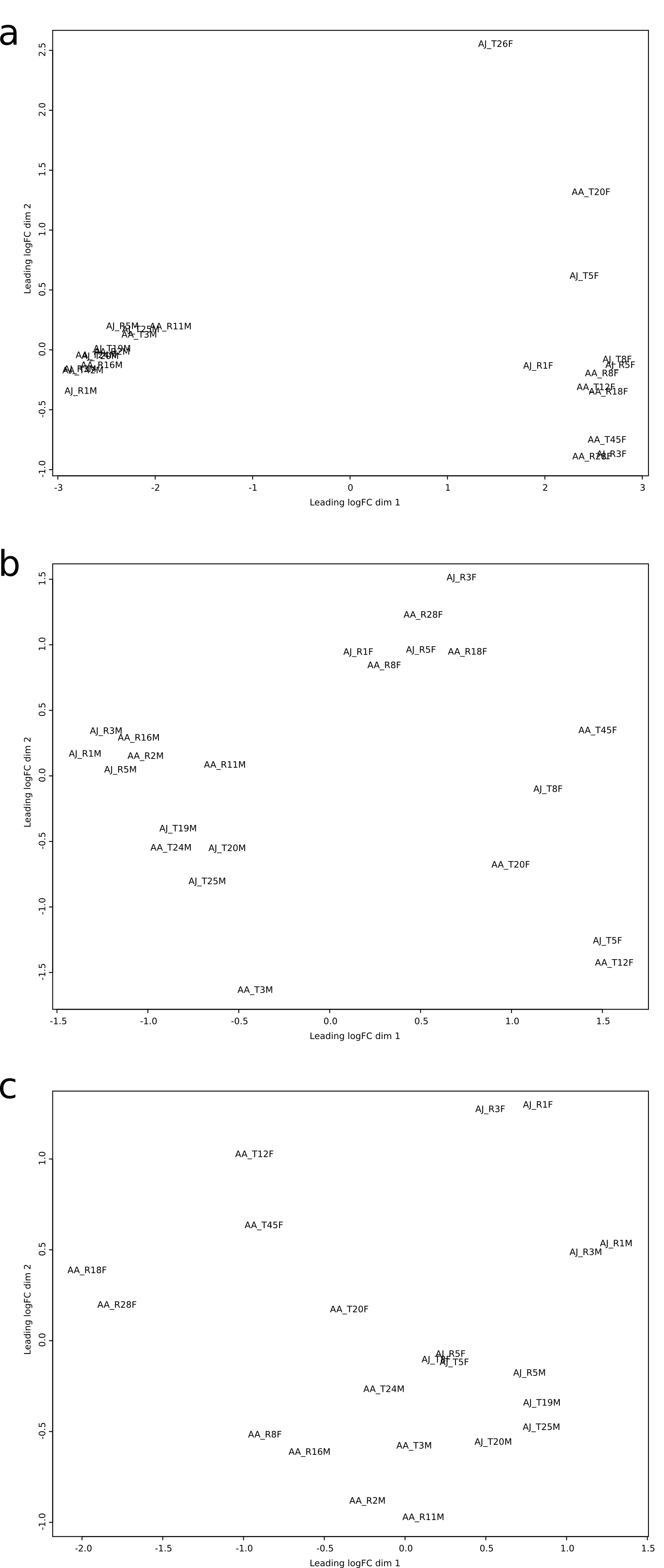

Supplement: Figure S1 — Multidimensional scaling of overall gene expression (A) identifies distinct clusters of male and female nematodes. Using the same technique on the subset of genes identified in generalized linear models for genes putatively differing for both nematode population (B) and experimental host species (C) does not generate robust clustering. [file peerj-02-684-s001.png]

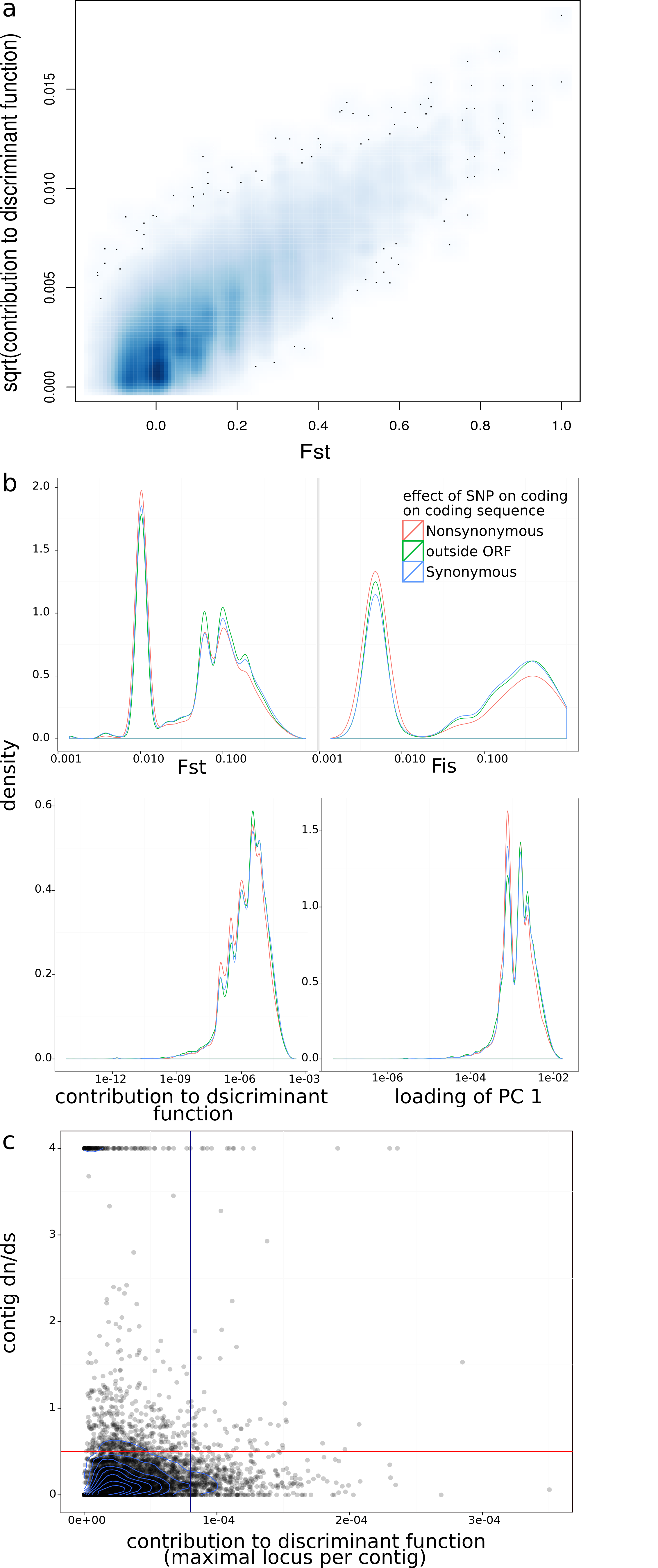

Supplement: Figure S2 — (A) depicts the high correlation of the contribution to the discriminant function between populations and fixation index Fst. (B) shows the density distributions of different measures of differentiation separately for SNPs with different effect on the protein sequence. Highly differentiating SNPs are enriched for synonymous and non-coding polymorphism over of non-synonymous differences. In (C) dn/ds is plotted against the maximal per gene differentiation (over loci), the green and blue line s give thresholds for “high differentiation” (maximal contribution to discriminant function > 0.8 × 10^−5^) and “positive selection” (dn/ds > 0.5), respectively. [file peerj-02-684-s002.png]
